# Sarcoma at the Site of a Transponder Implant in a Standing’s Day Gecko (*Phelsuma standingi*): A Case Report

**DOI:** 10.1155/crve/1376178

**Published:** 2026-03-03

**Authors:** Madeleine V. Thompson, Tessa Kell, Stefan M. Keller, Jasmine Rosario, Jenessa Gjeltema

**Affiliations:** ^1^ University of California Davis School of Veterinary Medicine, Davis, California, USA, ucdavis.edu; ^2^ Department of Pathology Microbiology and Immunology, University of California Davis School of Veterinary Medicine, Davis, California, USA, ucdavis.edu; ^3^ Sacramento Zoo, Sacramento, California, USA; ^4^ Department of Medicine and Epidemiology, University of California Davis School of Veterinary Medicine, Davis, California, USA, ucdavis.edu; ^5^ Karen C. Drayer Wildlife Health Center, University of California Davis School of Veterinary Medicine, Davis, California, USA, ucdavis.edu

## Abstract

A 26‐year‐old female Standing’s day gecko (*Phelsuma standingi*) was presented for evaluation of a mass on the right caudolateral trunk and was subsequently euthanized. Necropsy revealed a neoplastic mass surrounding a passive integrated transponder at the body wall. Histopathology of the mass was consistent with a sarcoma and was characterized by whorls of fusiform neoplastic cells invading the muscle, adipose, and bone of the coelomic wall. Given its proximity to the transponder and the degree to which it encompassed the device, the sarcoma was inferred to be transponder‐associated. However, further conclusions regarding a causal relationship between the transponder implant and the neoplastic mass cannot be drawn based on this single case. This report describes the clinical presentation and pathologic findings for a microchip‐associated neoplasia in a reptile.

## 1. Introduction

Microchips, or passive integrated transponders, are frequently used as a primary form of identification for animals under human care. Transponders are composed of a microchip and an antenna hermetically sealed and encapsulated in biocompatible glass. An electromagnetic field produced by a transponder reader supplies the energy required for the transmission of data from the transponder in the form of radio waves. Transponder‐associated sarcomas have been reported in a number of mammalian species, including cats, laboratory rodents, shrews, fruit bats, and mole rats [1–6]. Similar reports are generally lacking in the literature for reptiles, despite one study finding the prevalence of neoplasia in privately necropsied reptiles over the course of 9 years to be 9.8% [7]. In this study, soft tissue sarcomas comprised the majority of tumors diagnosed in snakes and lizards. Sarcomas are malignant tumors originating from mesenchymal tissues and are classified based on their cell of origin [8]. Previously published case reports of sarcomas in lizards include metastatic anaplastic sarcoma in a wild lace monitor (*Varanus varius*) [9]; anaplastic sarcoma, Sertoli cell tumors, soft tissue sarcoma, and periocular myxosarcoma in bearded dragons (*Pogona vitticeps*) [10–12]; chondroblastic osteosarcoma in related spiny‐tailed monitors (*Varanus acanthurus*) [13]; tracheal chondrosarcoma in a blue tegu (*Salvator merianae*) [14]; and oral fibrosarcoma in a black iguana (*Ctenosaura similis*) [15]. Of these reports, none were noted to be associated with transponders, and most authors considered the tumors to have occurred spontaneously. The current case describes the occurrence of a transponder‐associated sarcoma in a Standing’s day gecko (*Phelsuma standingi*).

## 2. Case Presentation

A 26‐year‐old female Standing’s day gecko weighing 36 g was evaluated for a mass on the right caudolateral trunk in December 2024. The gecko had been hatched and managed under human care at the Sacramento Zoo for its entire life. Over the 3 years prior to presentation, the gecko was maintained in a fiberglass enclosure measuring 91 cm in length, 81 cm in width, and 116 cm in height with a substrate mixture composed of organic bark, moss, coconut/plant fiber, and activated charcoal. Average enclosure temperature was maintained at 24°C–28°C with warmer basking locations provided. The average enclosure humidity was maintained around 65%, and UVB light (Zoo Med ReptiSun T8‐HO UVB Fluorescent Light; Zoo Med Laboratories Inc., 3650 Sacramento Drive, San Luis Obispo, California 93401) was provided on a 12‐h light cycle from the top of the enclosure with a measured intensity of 1.0–2.6 UVI at the animal’s preferred perching location. The diet consisted of Zoo Med Crested Gecko Food Tropical Fruit Flavor (Zoo Med Laboratories Inc., 3650 Sacramento Drive, San Luis Obispo, California 93401), Repashy Crested Gecko Food Classic Fruit Blend (Repashy Ventures, Inc., Avenida De La Plata, Oceanside, California 92056), crickets (*Acheta domesticus*) gut‐loaded with a high‐calcium diet, wax worms (*Galleria mellonella*), and mealworms (*Tenebrio molitor*) on a rotating basis.

The gecko had a history of cataracts, stifle osteoarthritis, occasional dysecdysis, chronic dehydration, nutritional secondary hyperparathyroidism, tail autotomy, and rare wounds requiring treatment from conspecific interactions. These conditions were static or resolved prior to presentation and were considered unrelated to the animal’s presenting problem. As treatment for chronic dehydration, the gecko had been receiving Lactated Ringer’s Solution (30 mL/kg; B. Braun Medical Inc., 824 12th Ave., Bethlehem, Pennsylvania 18018) subcutaneously once weekly for several years. A Trovan passive integrated transponder (Electronic ID Devices, LTD, PO Box 40227, Santa Barbara, California 93140) was placed subcutaneously at the right coelomic region just cranial to the right hind limb in 2007 (17 years prior to presentation) as a permanent form of animal identification. The animal had received preventive health examinations annually, with one having been performed 8 months prior to presentation. The mass was not present at this examination, and the location of the transponder was consistent with its location at the time of placement based on radiographs. Transponder function was confirmed at each exam using a Trovan transponder reader.

## 3. Clinical Findings

On physical examination, the gecko was quiet, alert, and responsive with moderate dehydration. She was mildly underconditioned with a body condition score of 3/9 and moderate diffuse muscle wasting. On the right caudolateral trunk, just cranial to the right hind limb, there was an approximately 2.5 × 1 cm firm mass (Figure 1a). The overlying integument was thickened, nodular, and was of a darker mottled gray compared to the surrounding skin. The skin discoloration extended to the ventral midline.

Figure 1Images of a Standing’s day gecko (*Phelsuma standingi*) diagnosed with a transponder‐associated sarcoma. (a) Thickened, lobulated, and discolored appearance of the skin overlying the sarcoma (for orientation, the head is toward the right). (b) Dorsoventral radiographic view of the animal prior to the development of the sarcoma. (c) Dorsoventral radiographic view of the animal at diagnosis of the sarcoma. Note the thickened skin (arrows) and increased space between the ribs and the transponder location. (d) Right lateral radiographic view of the animal prior to the development of the sarcoma. (e) Right lateral radiographic view of the animal at diagnosis of the sarcoma. The cranial border of the mass can be seen encircling the transponder (arrowheads).

**Figure:**
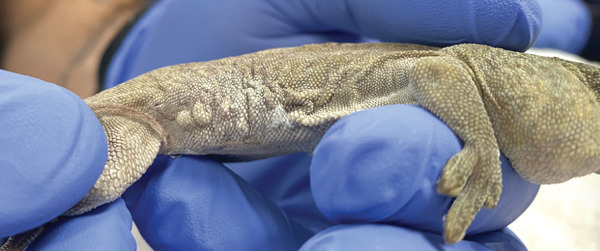
(a)

**Figure:**
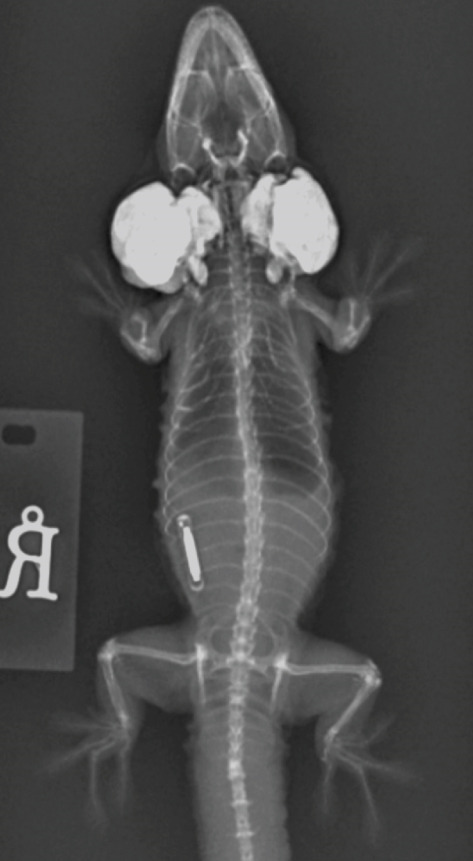
(b)

**Figure:**
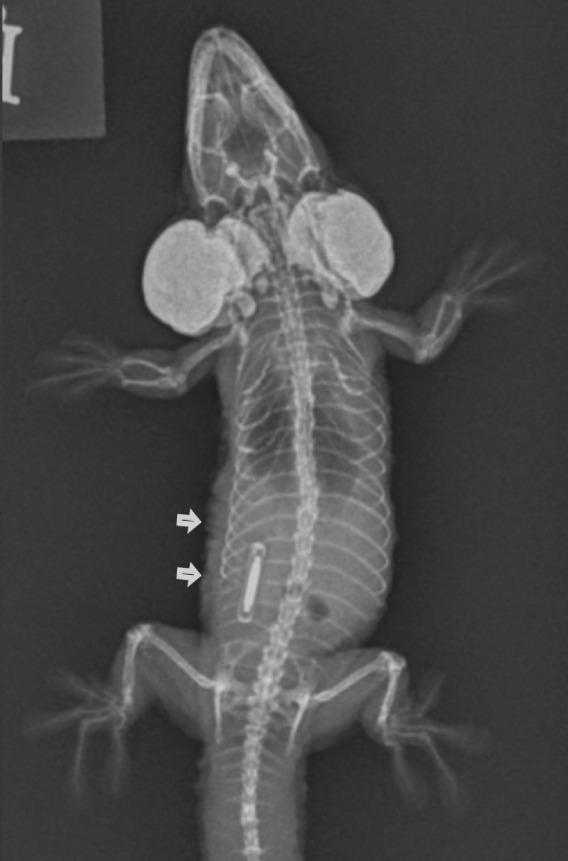
(c)

**Figure:**
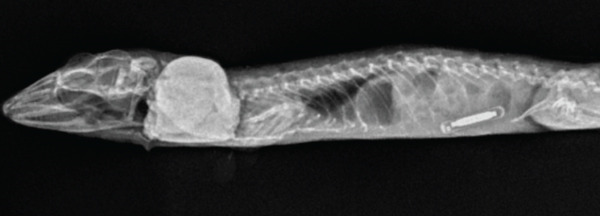
(d)

**Figure:**
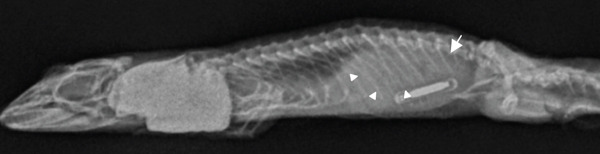
(e)

Whole‐body orthogonal radiographs (dorsoventral and right lateral projections) revealed a mass effect at the right caudal coelomic region just cranial to the right hind limb at the location of the animal’s transponder (Figure 1c,e). This radiographic mass correlated with the location of the mass observed during the physical exam. Radiographs showed regionally thickened skin and body wall at this location and dorsomedial displacement of the transponder compared to historical radiographs (Figure 1b,d).

An estimated white blood cell count and differential were performed from a blood smear and revealed a leukopenia of 1750 cells/*μ*L (historically 3500–5280 cells/*μ*L) with a relative heterophilia of 1590 cells/*μ*L (historically 490–790 cells/*μ*L) and toxic morphologic changes present for some heterophils. Due to a lack of reference values for this species, results were compared to historical values obtained from this animal during routine exams over the last 10 years. Sample volume was insufficient for further analysis.

Ultrasound of the mass was performed using a high‐frequency linear transducer, which revealed an expansion of hypoechoic tissue extending from the body wall through intercostal spaces and into adjacent subcutaneous tissues.

Primary differentials included neoplasia or severe infection at the site. Given the gecko’s advanced age, small size, and multiple comorbidities, her prognosis was classified as poor, and a palliative approach was elected. She was treated with tramadol (5–10 mg/kg orally every 24 h) and given empirical antibiotic therapy with trimethoprim/sulfamethoxazole (26 mg/kg orally every 24 h for 14 days). Due to a declining quality of life, she was euthanized 3 weeks later.

## 4. Pathologic Findings

At necropsy, the right caudodorsal body wall, just cranial to the right coxofemoral joint, had a 1.2 × 1 × 0.8 cm irregularly marginated, firm region that was overlain by a 1.5 × 1 cm ulcerated region of skin. On section, the right caudodorsal body wall was thickened up to 8 mm by an unencapsulated, irregularly marginated, poorly demarcated, expansile and infiltrative, pale tan‐to‐pink, firm mass that surrounded and encompassed a passive integrated transponder device (Figure 2a). The mass displaced the coelomic viscera to the left side of the body cavity. There were several fibrous, firm, tan adhesions extending between the liver capsule and the right caudal body wall at the location of the mass, and there was approximately 0.5 mL of watery, opaque, pale red fluid within the coelomic cavity. The thoracolumbar portion of the vertebral column, at the level of the body wall mass, was laterally displaced approximately 5 mm to the left and formed an S‐shaped curve.

Figure 2Gross and histologic images of a transponder‐associated sarcoma within the body wall of a Standing’s day gecko (*Phelsuma standingi*). (a) Postformalin fixation. The mass was incised, exposing the transponder device located centrally within the mass. (b) (HE) The oval, clear space located centrally within the mass is the location where the transponder device was removed from. The mass lifted the overlying epidermis and dermis and invaded through the skeletal muscle, adipose, and bone of the coelomic wall, surrounding and isolating individual rib bones. (c) (HE) The neoplasm invaded through the skeletal muscle, isolating individual atrophied myocytes. (d) (HE) The neoplasm was composed of sheets, streams, and loose whorls of fusiform cells supported by a fine fibrovascular stroma.

**Figure:**
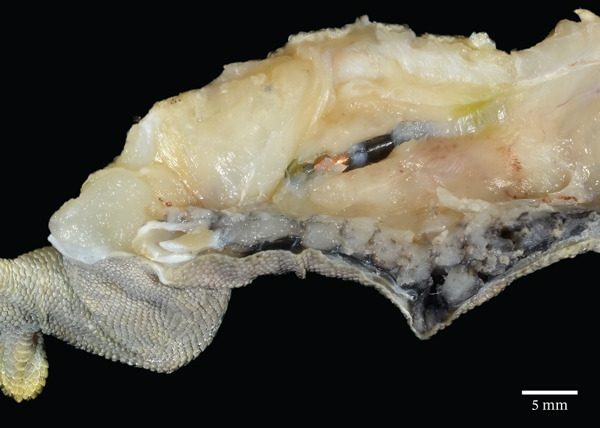
(a)

**Figure:**
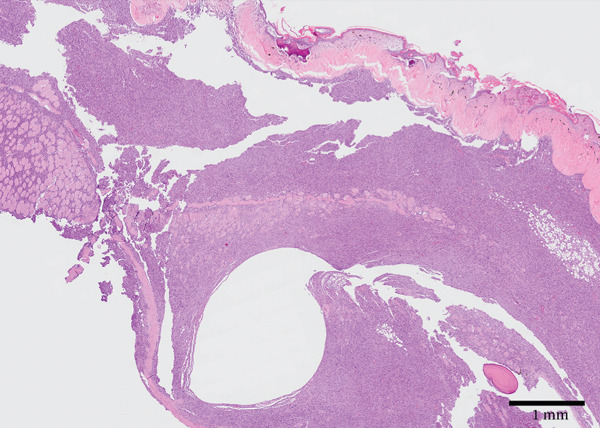
(b)

**Figure:**
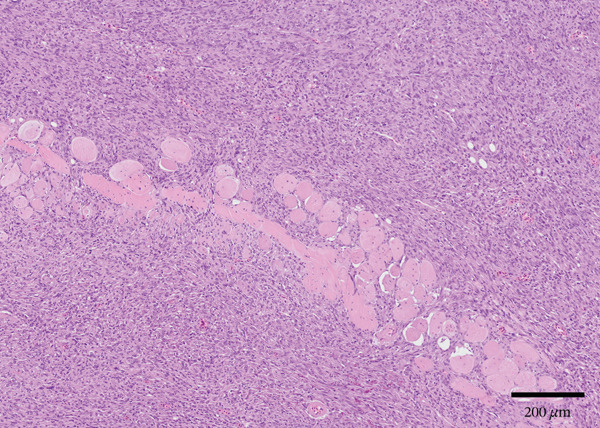
(c)

**Figure:**
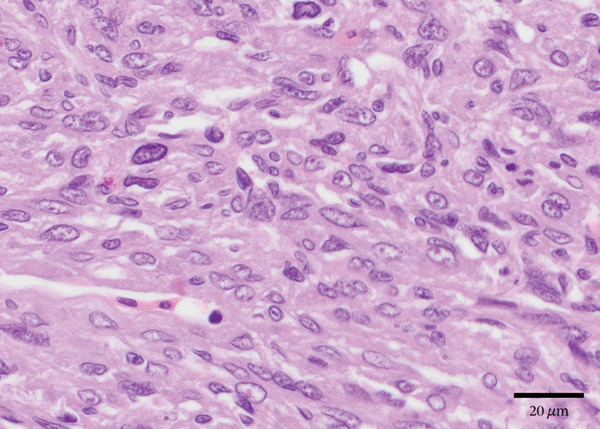
(d)

Microscopic examination of the body wall mass revealed effacement of the coelomic wall by an unencapsulated, moderately well‐demarcated, expansile and infiltrative, densely cellular mass that surrounded a central, round, approximately 2‐mm‐diameter clear space from which the transponder was removed (Figure 2b). The mass was composed of sheets, streams, and loose whorls of fusiform cells supported by a fine fibrovascular stroma that lifted the overlying epidermis and dermis and invaded through the skeletal muscle, adipose, and bone of the coelomic wall, surrounding and isolating individual atrophied myocytes and adipocytes, as well as rib bones with bony modeling (Figure 2c). The neoplastic cells frequently had indistinct borders, moderate amounts of pale basophilic cytoplasm, and one to occasionally two oval‐to‐elongate nuclei with variably condensed chromatin, as well as one to two variably visible, eosinophilic nucleoli (Figure 2d). Anisocytosis and anisokaryosis were moderate, and mitotic figures were rare (2 per 10 high‐power fields). The neoplasm extended through the serosa, although not visible grossly, and there was a single section of small intestine with a focus of neoplastic cells invading the serosa. Decalcified sections of the thoracolumbar vertebrae from the region with scoliosis revealed extensive invasion of the neoplasm into the right epaxial and hypaxial muscles without invasion into the vertebrae or compression of the spinal cord.

## 5. Discussion

Microchips have been a cornerstone of identification systems for animals in a variety of settings since their introduction in the 1980s. They were quickly adopted by institutions housing species of laboratory animals for which other methods of identification were not feasible. Common alternatives such as tags, banding, and toe clipping, for instance, cannot be reliably performed in common laboratory species such as axolotls or *Xenopus* spp. [16]. For reptiles, common methods include morphologic modifications (i.e., toe clipping, shell notching, and branding), photo identification, banding, and color marking [17].

While there is a potential risk of tumor development associated with implanted transponder devices, they remain a reliable method of primary identification in many exotic species. Of the animals that receive transponder implants, associated neoplasms are a relatively uncommon occurrence. Thus far, transponder‐associated tumors have only been reported in mammalian species, and the underlying mechanism is not fully understood. The majority of these tumors are different types of sarcomas [1–6, 18], as well as one amelanotic melanoma [6] and two adenocarcinomas [3]. In the present study, further subtyping of the neoplasm was not pursued due to the lack of validated immunohistochemical markers in this species.

The sarcoma described in this report arose from the region of transponder implantation and fully encompassed the device, suggesting that the transponder may have contributed to tumor formation. However, a causative relationship between transponder placement and neoplasm development remains speculative since it cannot be substantiated without controlled studies.

A proliferative response to foreign body implantation may serve as a precursor to neoplastic transformation. A prior study using transgenic mice heterozygous for the tumor suppressor gene *p53* revealed a 10% prevalence of microchip‐associated sarcomas [18]. In contrast, several studies using nontransgenic animals have only demonstrated nonneoplastic proliferative changes following subcutaneous transponder implantation. Given that transponders are not composed of known carcinogenic materials, the higher incidence of neoplasia in *p53* heterozygous mice may indicate a distinct oncogenic mechanism. It is possible that the initial proliferative response to implantation acts as a trigger for neoplastic transformation, particularly in genetically susceptible individuals. In this context, *p53* heterozygous mice may model a rare process that could occur in other species following transponder implantation.

Tissue reaction to foreign body implantation has been described in various species, with one paper citing a chronic fibrotic process as an inciting event for tumorigenesis [2]. Seven cases of extraskeletal osteosarcomas associated with intralesional foreign material have been reported in dogs: three associated with surgical gauze or sponge material, two with salivary‐associated mucus accumulation, one with keratin from a benign follicular tumor, and one associated with cotton‐based toy fragments [19]. Feline injection site sarcomas are anatomically associated with sites of rabies and feline leukemia virus (FeLV) vaccinations and are locally aggressive. The pathogenesis of these tumors is not known but is thought to be associated with an inflammatory reaction to vaccine components. Aluminum‐based adjuvants in particular have been proposed as the main inflammatory nidus. A retrospective study of cases from the Swiss Feline Cancer Registry showed a reduction of fibrosarcoma diagnoses from 20% to 11% from 2009 to 2014 following the introduction of nonadjuvanted FeLV vaccines in Switzerland in 2007, suggesting a relationship between adjuvanted vaccines and fibrosarcoma development [20]. In humans, sarcomas are considered a rare postsurgical complication of metal orthopedic implants [21].

Antemortem diagnostics are limited by the small size of many reptilian species, and exhaustive classification of neoplastic masses is not always practical or performed due to the lack of validated immunohistochemical antibodies. This report describes a transponder‐associated tumor in a nonmammalian species and provides additional evidence that the majority of these tumors are mesenchymal in origin. Neoplasia should be considered a differential diagnosis for any mass arising near a transponder site in a reptilian species. Given the high volume of transponders placed into animals in a variety of settings, further research on the mechanisms driving this process is warranted.

## Author Contributions

M.V.T. and T.K. contributed equally to this work.

## Funding

No funding was received for this manuscript.

## Conflicts of Interest

The authors declare no conflicts of interest.

## Data Availability

The data that support the findings of this study are available on request from the corresponding author. The data are not publicly available due to privacy or ethical restrictions.
